# Effects of Chronic Exposure to Arsenic and Estrogen on Epigenetic Regulatory Genes Expression and Epigenetic Code in Human Prostate Epithelial Cells

**DOI:** 10.1371/journal.pone.0043880

**Published:** 2012-08-27

**Authors:** Justin N. Treas, Tulika Tyagi, Kamaleshwar P. Singh

**Affiliations:** Department of Environmental Toxicology, The Institute of Environmental and Human Health (TIEHH), Texas Tech University, Lubbock, Texas, United States of America; Roswell Park Cancer Institute, United States of America

## Abstract

Chronic exposures to arsenic and estrogen are known risk factors for prostate cancer. Though the evidence suggests that exposure to arsenic or estrogens can disrupt normal DNA methylation patterns and histone modifications, the mechanisms by which these chemicals induce epigenetic changes are not fully understood. Moreover, the epigenetic effects of co-exposure to these two chemicals are not known. Therefore, the objective of this study was to evaluate the effects of chronic exposure to arsenic and estrogen, both alone and in combination, on the expression of epigenetic regulatory genes, their consequences on DNA methylation, and histone modifications. Human prostate epithelial cells, RWPE-1, chronically exposed to arsenic and estrogen alone and in combination were used for analysis of epigenetic regulatory genes expression, global DNA methylation changes, and histone modifications at protein level. The result of this study revealed that exposure to arsenic, estrogen, and their combination alters the expression of epigenetic regulatory genes and changes global DNA methylation and histone modification patterns in RWPE-1 cells. These changes were significantly greater in arsenic and estrogen combination treated group than individually treated group. The findings of this study will help explain the epigenetic mechanism of arsenic- and/or estrogen-induced prostate carcinogenesis.

## Introduction

Prostate cancer is the second leading cause of cancer death in American men and both endogenous and exogenous factors are involved in prostate carcinogenesis [1]. Hormonal imbalance occurring with old age is associated with the high incidence of prostate cancer (1 in 100) in men over the age of 65 [2]. Prolonged exposure to elevated levels of natural estrogen, 17β-estradiol, or xenoestrogen plays significant role in development and growth of prostate and induction of prostate cancer [3], [4]. For example, fetal exposure to the xenoestrogens ethinylestradiol and bisphenol A increase the size of prostate at adult age in mice [5]. Neonatal exposure to 17β-estradiol (E2), or synthetic estrogen, diethylstilbestrol, causes increased incidence of prostate intraepithelial hyperplasia (PIN), a preneoplastic lesion in the prostate of mice [6]. Moreover, exposure to higher levels of estrogen during early developmental period predisposes to prostate cancer development later in life [6].

Similarly, epidemiological and experimental studies suggest an association between prostate cancer and chronic exposure to inorganic arsenic [7]. Humans are exposed to arsenic primarily through drinking water and inhalation of contaminated dust from coal burning. Increased prostate cancer incidence and mortality in certain US population that were exposed to higher levels of arsenic through their drinking water, further indicates a strong association between arsenic exposure and prostate cancer [8]. Fetal and/or neonatal exposure to arsenic causes cancers, including that of the prostate at adulthood in mice [9]. Effects of arsenic on the expression of several genes and their role in neoplastic transformation of cells are well documented [10], [11]. However, the mechanism by which arsenic and/or estrogen exposure contributes to the development of prostate cancer is not well understood. Moreover, the effects of the combined exposures to these two chemicals on prostate epithelial cells are not known.

Chromatin remodeling by epigenetic reprogramming controls the regulation of gene expression and has important implications in development of human cancers [12], [13]. For example, DNA methylation can contribute to carcinogenesis in several ways including loss of imprinting, generation of chromosomal instability, re-activation of transposons, and activation of normally methylated oncogenes [14]. Post-translational modifications of histones and DNA methylation changes are two important epigenetic mechanisms that control active or passive chromatin structure and gene expression [15]. Alterations in DNA methlyation and histone modification patterns have been extensively reported in cancerous tissue [12]. Therefore, evaluation of the effects of arsenic and estrogen on expression of these genes could be helpful in understanding the mechanism of arsenic and/or estrogen –induced prostate cancer development.

DNA methylation plays an important role in maintaining normal expression of genes and genomic stability by controlling expression of tumor suppressor genes and repression of repetitive sequences that may otherwise cause genomic instability [16]. DNA methylation is regulated by DNA methyltransferases (DNMTs) and altered expression of DNMTs has been reported in human tumor tissue [17]. Normal DNA methylation patterns are maintained by DNMT1 [13], whereas *DNMT3a* and *3b* nondiscriminately methylate unmethylated cytosine or hemimethylated cytosine in the genome [18]. Additionally, methyl binding proteins, *MBD1-4* and *MeCP2*, also play an important role in epigenetic programming by recognizing and binding to CpG islands [13], [19]. Post-translational modifications, such as, acetylation and methylation of histones are another epigenetic mechanism for regulation of gene expression [20]. Deacetylation of the histone at N-terminal tail by histone deacetylases (HDACs) causes compact chromatin structure leading to transcriptional repression of genes [20], whereas its acetylation by histone acetyltransferases (HATs) results in a loose chromatin structure that facilitates increased gene transcription [21]. Similarly, histone methylation mediated by histone methyltransferases (HMTs) also plays an important role in gene activation and suppression depending upon target amino acids for methylation [12], [22].

Accumulating evidence strongly indicates that prostate cancer is driven by accumulation of genetic and epigenetic aberrations [23]. Apart from global hypomethylation, specific genes like *GSTP1*, *APC*, *MDR1*, *14-3-3σ*, *GPX3* have been shown to be inactivated by hypermethylation in prostate cancer [24]. Another study has shown that the frequent loss of *E-cadherin* in prostate cancer cell lines results from hypermethylation [25]. Similar findings have been demonstrated in a US population-based study which revealed hypermethylation of *CD44* and *E-cadherin* genes in prostate cancer [26]. Several other genes involved in various functions, such as, hormonal response (*AR*, *ESR1*, *ESR2*, *RARβ)* cell cycle (*CCND2*, *CDKN2A*), tumor cell invasion (*CAV1*,*CDH1*,*CDH13*,*LAMA3*), and signal transduction (*DAPK1*, *DAB2IP*, *RASSF1*) are also affected by DNA hypermethylation in prostate cancer [27].

Besides the genetic changes of DNA damage and mutations in both nuclear and mitochondrial DNA [28], several reports suggest that arsenic and/or estrogen causes epigenetic changes of DNA methylation and histone modifications in prostate as well as other target organs. For example, aberrant genomic DNA methylation was observed in human prostate epithelial cells exposed to arsenic [7]. Inorganic arsenic -induced malignancy in human prostate epithelial cell line is associated with genomic DNA hypomethylation [29]. Dose-dependent hypermethylation at promoter region of tumor suppressor genes like p53 and p16 has been observed due to exposure to inorganic arsenic *in vivo* and *in vitro* [30]. Hypermethylation of *DAPK* gene was observed in urothelial carcinoma patients living in arsenic –contaminated areas as well as in immortalized human uroepithelial cells exposed to arsenic [31], [32]. Overexpression of *ESR1* gene resulting from arsenic induced promoter region hypermethylation was observed in hepatocarcinogenesis [31], [33]. Sodium arsenite exposure significantly increases global histone acetylation through HDAC inhibition [34]. Despite these evidences of epigenetic aberrations in prostate carcinogenesis, the effects of arsenic on the expression of epigenetic regulatory genes are not well understood.

Additionally, the co-exposure to arsenic and estrogen (elevated level of endogenous estrogen or exogenous estrogenic chemicals) is a likely scenario that may potentiate the adverse effects leading to prostate carcinogenesis. Therefore, evaluation of the effects of these two chemicals, alone as well as in combination, will provide a better understanding of the molecular mechanism(s) through which these chemicals exert their carcinogenic effects in prostate epithelial cells. It is in this context that we have analyzed, for the first time, the effects of arsenic and estrogen, both alone and in combination, on the expression of epigenetic regulatory genes and the consequences on DNA methylation and histone modification patterns in human prostate epithelial cells.

## Methods

### Chemicals

Sodium meta-arsenite, NaAsO_2_ (As)_,_ and 17β-estradiol (E2) were purchased from Sigma Chemical Company. Trizol for RNA isolation was purchased from Invitrogen, Inc. The limit for arsenic in drinking water is 10 ppb as set by Environmental Protection Agency (EPA) and International Agency for Research on Cancer (IARC) [7]. Therefore, the 100 pg/mL (0.1 ppb) dose is 100 times less than the EPA limit and the 100 ng/mL (100 ppb) dose is 10 times greater than the EPA limit. Blood serum levels of E2 range from 25 pg/mL to 40 pg/mL in men [35]. Therefore, 100 pg/mL dose of E2 used in this study is closer to serum E2 levels in men and 100 ng/mL dose is higher than serum levels of E2 in men. These lower doses of As and E2 were in the physiological range and the higher doses were included to evaluate the effects of elevated levels of these chemicals.

### Cell Culture

Human prostate epithelial cells (RWPE-1) were purchased from ATCC and propagated in keratinocyte serum free medium supplemented with human epithelial growth factor and bovine pituitary extract. RWPE-1 cells are immortalized but non-tumorigenic derived from the human prostate. Cultures were incubated at 37°C in a humidified atmosphere containing 5% CO_2_.

### Arsenic and Estrogen Treatment of Cells

Actively growing cells were seeded in 25 cm^2^ culture flask and allowed to attach. Cultures were maintained and treated in triplicate. Once the cells had become approximately 25% confluent the media was replaced with fresh media and cells were treated with the 100 pg/mL and 100 ng/mL of As and E2, both individually as well as in combination of these two chemicals at respective concentrations (100 pg/mL of each and 100 ng/mL of each). After every 6 days, cells grown to near confluence (70–80%) were sub-cultured and the process was repeated for a period of six months. Parallel cultures grown and treated with vehicle (DMSO) only were used as passage-matched controls.

### DNA and RNA Extraction

For DNA and RNA extraction, cells were seeded in 75 cm^2^ culture flasks and allowed to reach near sub-confluency (70–80%). Total genomic DNA was isolated by SDS/proteinase K digestion and phenol-chloroform extraction method as described earlier [28]. The total RNA from each treatment and control group was isolated using Trizol reagent (Invitrogen, Inc.). DNA and RNA were quantified spectrophotometrically and their quality was checked on 1% agarose gels by ethidium bromide staining.

Methylation-Sensitive Random Amplified Polymorphic DNA (MS-RAPD)-PCR amplification.

Methylation-sensitive arbitrary primed polymerase chain reaction (AP-PCR) also known as random amplified polymorphic DNA (RAPD) was performed to detect DNA methylation changes at global genome level. The methylation sensitive-RAPD (MS-RAPD) technique involves digestion of genomic DNA with methylation-sensitive (e.g. *Hpa*II) and –insensitive (e.g. *Msp*I) restriction enzymes followed by RAPD-PCR amplification [36], [37]. Since the primers are smaller in length (10 bp), these individual primers bind at multiple sites (depending on the number of complementary sequence in the genome) in genomic DNA at low annealing temperatures and produce multiple PCR amplification products of various sizes. The MS-RAPD-PCR products are then resolved either on agarose or polyacrylamide gel to detect genomic DNA regions associated with hypo- and hypermethylation.

Total genomic DNA was used to perform MS-RAPD by following earlier published methods [36], [38]. An aliquot of 2 µg DNA was digested with methylation sensitive restriction enzymes *MSPI* and *HPAII*. Using restriction enzyme digested, as well as undigested DNA, the RAPD-PCR amplifications were performed in 25 µL of reaction mixture containing 2.5 µL of 10 X enzyme assay buffer, 100 µM each of dATP, dCTP, dGTP, dTTP (Applied Biosystems, Foster City, CA), 100 nM of random primer (10- bp), 2.5 mM MgCl_2,_ 0.5 unit of AmpliTaq DNA polymerase (Applied Biosystems, Foster City, CA) and 75 ng of genomic DNA as template. The amplifications were performed in a DNA thermal cycler (GeneAmp PCR System 2700) programmed for 45 cycles as follows: 1^st^ cycle (3.5 min at 92°C, 1 min at 34°C, 2 min at 72°C), next 44 cycles (1 min at 92°C, 1 min at 34°C, 2 min at 72°C) followed by a final extension cycle of 15 min at 72°C. The PCR products were resolved on 1.5% agarose gel and visualized by ethidium bromide staining.

### Quantitative Real-time PCR

One-step RT-PCR kit with SYBR green was used for amplification of total RNA (200 ng) following the manufacturer’s protocol (Bio-Rad). Single-step RT-PCR amplifications were performed using Rotor Gene (Corbette Research Inc) real-time PCR machine programmed for reverse transcription at 50°C for 15 min, denaturation and RT enzyme inactivation at 95°C for 5 min, followed by 40 cycles each containing 10 seconds for denaturation at 95°C and 30 seconds for annealing and extension at 60°C. Specificity of the PCR products was confirmed by melt curve analysis. Data were normalized to Ct values of *GAPDH* from the same sample and the fold-changes in expression of each gene were calculated by using the ΔΔCt method [39]. A non-template control was included in each experiment. Primer sequences used are given in Table 1.

**Table 1:** A list of forward and reverse primers sequence of the genes used in gene expression analysis by quantitative real time PCR.

Primer	Forward primer (5′ to 3′)	Reverse Primer (5′ to 3′)	Product size (bp)
GAPDH	GGTGGTCTCCTCTGACTTCAACA	GTTGCTGTAGCCAAATTCGTTGT	116
DNMT1	AGCAAGAAGTGAAGCCCGTA	TGAACGCTTAGCCTCTCCAT	115
DNMT3a	CCTGAAGCCTCAAGAGCAGT	AGCCAAGTCCCTGACTCTCA	143
HDAC1	TGGAAATGTATCGCCCTCAC	TCTCTGCATCTGCTTGCTGT	128
HDAC3	TGGCTTCTGCTATGTCAACG	GACCCGGTCAGTGAGGTAGA	136
HDAC2	CTCCCAAAGTGCTGGGATTA	CCCATCTGGCATCTAAAGGA	138
HDAC7	CAGCTTTTTGCCTCCTGTTC	TTTCGGAGCAGTGGATTCTT	144
MeCP2	ATCTTCTGTTGGGTGGCATC	CTCTTTCGCCATCACTGTCA	128
MBD1	CAACCGGGAACAGAGAATGT	CTCCGTTCACACTTGCAGAA	147
MBD4	CAGGCAAAATGGCAATACCT	GTTTTTGCCCGAAGATCGTA	136
HAT1	GGTGATTCGTCCTTCCTCA	GCCAGTTTCTTCTCCACTGC	112
HMT1	TCCTCGTGCTGTGTGAAGAC	AAACGGTGAGAGATGCTGCT	127

### Western Blot Analysis

Total cellular protein lysates were prepared and electrophoretically separated on a 10% SDS-PAGE gel. After electrophoresis, proteins were transferred from gel onto nitrocellulose membrane, and then the membranes were blocked with 5% non-fat dried milk in 1X PBS containing 0.05% Tween 20, overnight at 4°C. Blots were incubated with diluted primary antibody for 1 h at room temperature. Based on the reaction efficiency of the primary antibodies, the dilutions of 1∶100 of *GAPDH* (Santa Cruz, Cat # sc-25778), H3K4me3 1:500 (Millipore Cat # 04–745), p-Ac-H3 (S 11/K 15) 1∶500 (Santa Cruz Cat # sc-33361), and 1∶400 of *MBD4* (Santa Cruz, cat #SC-10753) were used. Membranes were washed three times each for 10 minutes with washing buffer (1X PBS containing 0.05% Tween 20) and then incubated with appropriate horseradish peroxidase-conjugated secondary antibody at a dilution of 1∶1000 for 1 h at room temperature. Membranes were again washed three times each for 10 minutes and signals of the specific protein bands were then visualized by using an enhanced chemiluminescence detection system (Amersham, NJ). The band intensity was quantified by Image J software. The value of control was converted to 1 and the values of the treatment groups were accordingly converted with respect to control. Using these values, a histogram for band intensity was plotted.

### Statistical Analysis

To determine whether the differences observed were statistically significant, a t-Test (two-tailed, paired samples for means, and hypothesized difference of 0) was performed on the data. An ANOVA was performed to determine if the source of variation in the data was between or within treatment groups. Alpha was set at 0.05 for all statistical tests and data with p<0.05 were considered as significantly different.

## Results

**Figure 1:**
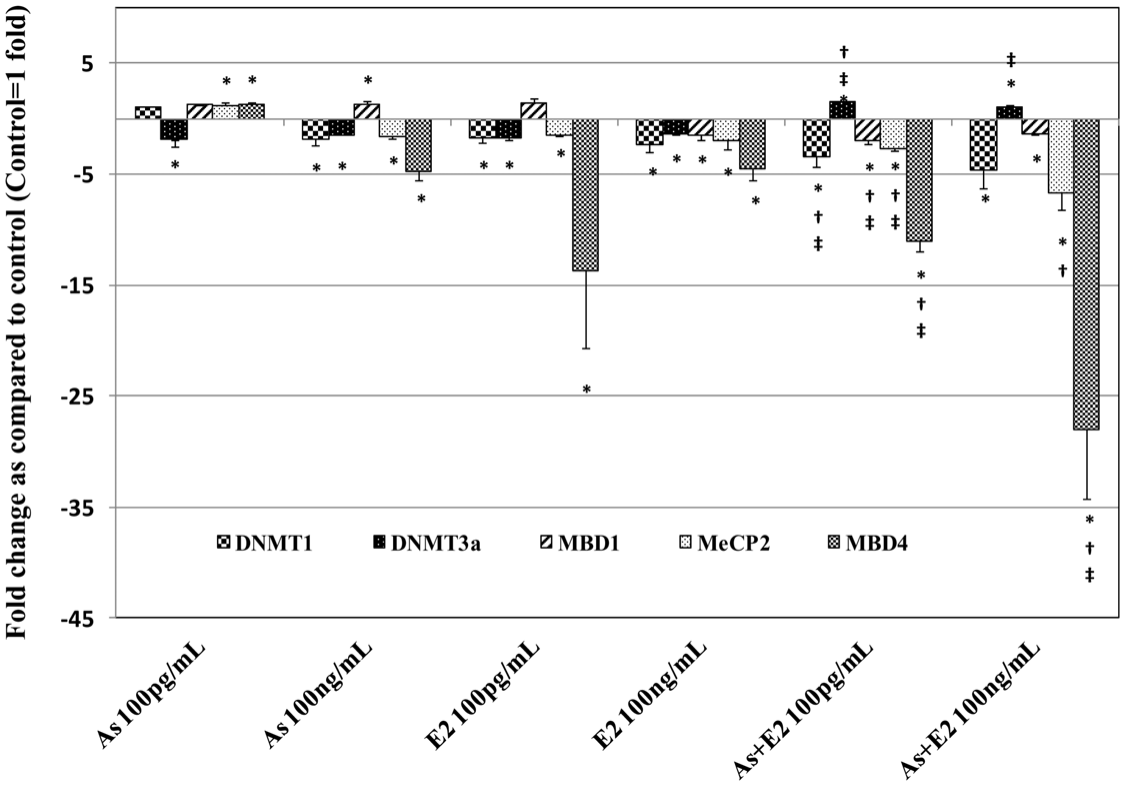
Real-time quantitative reverse transcription PCR analysis of gene expression of DNA methylation related genes. Total RNA isolated from RWPE-1 cells with chronic exposure to arsenic and/or estrogen was used to perform one-step real-time quantitative reverse transcription PCR as described in materials and methods. Cycle threshold value (Ct value) of each gene was normalized to the Ct value of housekeeping gene GAPDH obtained from the same sample. The gene expression in fold- change was calculated and histogram was plotted using the means of triplicate values. Statistically significant change in gene expression in treated groups as compared to the vehicle treated control is indicated by an *. Similarly, † indicates a significant difference (*p<0.05*) between As alone and As in combination with E2. ‡ indicates a significant difference (*p<0.05*) between E2 alone and E2 in combination with As.

**Figure 2:**
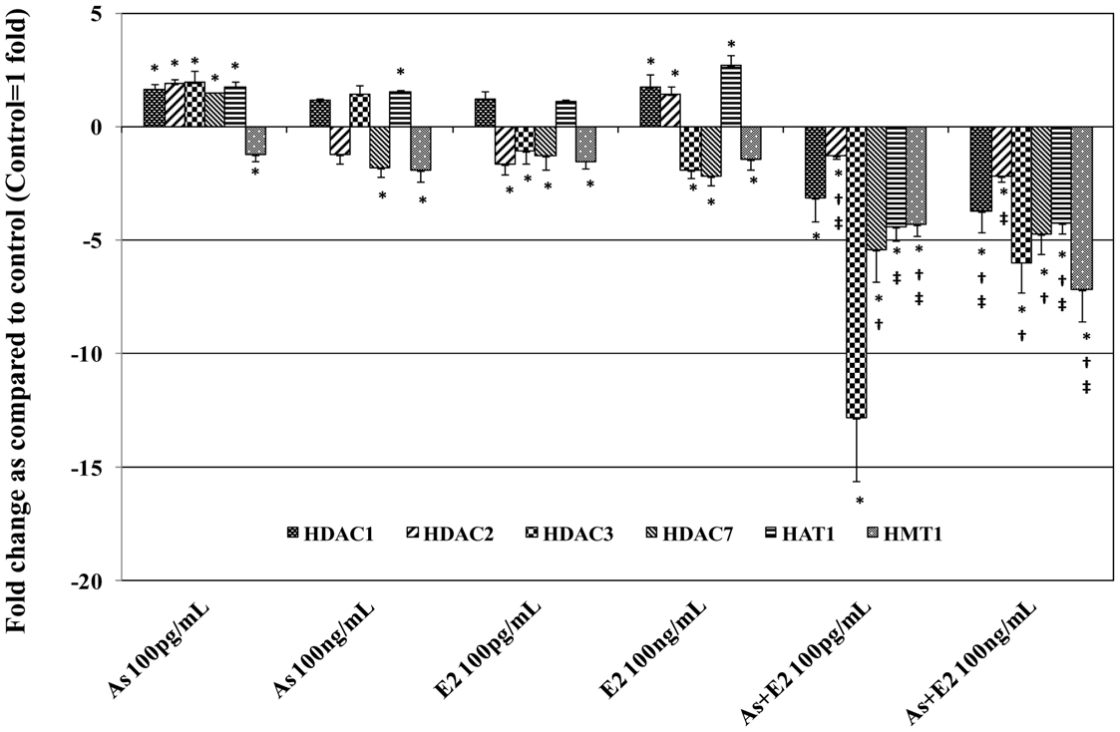
Real-time quantitative reverse transcription PCR analysis of gene expression of histone modifications- related genes. Total RNA isolated from RWPE-1 cells with chronic exposure to arsenic and/or estrogen was used to perform one-step real-time quantitative reverse transcription PCR as described in materials and methods. Cycle threshold value (Ct value) of each gene was normalized to the Ct value of housekeeping gene GAPDH obtained from the same sample. The gene expression in fold- change was calculated and histogram was plotted using the means of triplicate values. Statistically significant change in gene expression in treated groups as compared to the vehicle treated control is indicated by an *. Similarly, † indicates a significant difference (*p<0.05*) between As alone and As in combination with E2. ‡ indicates a significant difference (*p<0.05*) between E2 alone and E2 in combination with As.

A summary of gene expression changes in RWPE-1 cells exposed to As and E2 alone and in combination is given in Figures 1 and 2. A detailed description of the changes in gene expression as well as DNA methylation and histone modifications is categorized under the following sub-headings.

### 1. Genes Involved in DNA Methylation

To understand the role of As and E2 induced changes in the expression of DNA methyltransferases and methyl binding proteins involved in mediating the carcinogenic effects of these chemicals, gene expression changes at transcript level of *DNMT1* (for maintenance of DNA methylation), *DNMT3a* (de novo DNA methylation), and methyl binding proteins (*MBD1, MBD4*, and *MeCP2*) was determined by quantitative real time PCR. Details of the results in As, E2 and their combination treated groups are as follows.

#### 1.1. Effects of chronic as exposure on the expression of DNA methyltransferases and methyl binding proteins

The results of changes in expression of genes that are involved in DNA methylation (*DNMT1, DNMT3a, MeCP2, MBD1,* and *MBD4)* are presented in Figure 1. Expression of *DNMT1, MBD1, MBD4* and *MeCP2* was increased whereas the expression of *DNMT3a* was decreased in cells exposed to As 100 pg/mL as compared to untreated passage matched control (Figure 1). However, expression of these genes decreased in cells exposed to 100 ng/mL of As except for *MBD1* which exhibited an increase in expression (Figure 1). The changes observed in the expression of all these genes at both 100 pg/mL and 100 ng/mL were statistically significant (*p*<0.05) except the expression of *DNMT1* in 100 pg/mL treated cells were insignificant (*p*>0.05).

#### 1.2. Effects of chronic E2 exposure on expression of DNA methyltransferases and methyl binding proteins

Significantly (*p*<0.05) decreased expression of *DNMT1, DNMT3a, MBD4* and *MeCP2*, whereas an increased expression of *MBD1* was observed in cells exposed to 100 pg/mL of E2. Expression of these genes was significantly decreased in cells exposed to 100 ng/mL of E2. Among all the genes analyzed, the maximum reduction in expression was observed in *MBD4* with 13.71 fold decrease in 100 pg/mL and 4.52 fold decrease in 100 ng/mL of E2 treated cells (Figure 1).

#### 1.3. Effects of chronic co-exposure to as and E2 on the expression of DNA methyltransferases and methyl binding proteins

Combined exposure to As and E2 had a relatively greater effect on the expression of all five genes analyzed except *DNMT3a*, and *MBD1* as compared to single chemical exposure (Figure 1). For example, decreased expression of *DNMT1* by 3.39 fold in 100 pg/mL and 4.55 fold in 100 ng/mL of combination of As and E2 treated cells was much greater than decreased expression observed in cells exposed to these two chemicals individually (Figure 1). Combined exposure resulted in a greater downregulation of *MBD4*, with 11.02 fold in 100 pg/mL, and 27.96 fold in 100 ng/mL, of As and E2 combination treated cells (Figure 1). Changes observed in the expression of genes in combination treatment were statistically significant p<0.05 as compared to their control.

### 2. Genes Involved in Histone Modification

To understand the effect of As and E2 on histone modifications, the expression of representative genes for class 1 histone deacetylases (*HDAC* 1, 2, and 3) and class II histone deacetylase (*HDAC7*) as well as (*HAT1*) and (*HMT1*) were determined by quantitative real-time PCR.

#### 2.1. Effects of chronic exposure to as on the expression of histone modifying genes

The expression of *HDAC1, 2*, *3,* and *7,* as well as *HAT1,* were increased whereas expression of *HMT1* was decreased in cells exposed to 100 pg/mL of As (Figure 2). However, decreased expression of *HDAC2,* and *7,* as well as *HMT1*, and increased expression of *HDAC1, 3,* and *HAT1* were observed in cells exposed to 100 ng/mL of As. This increase in the expression of *HDAC1, HDAC3, HAT1* was relatively greater in cells exposed to lower dose (100 pg/mL) of As than the cells exposed to a higher dose (100 ng/mL) of As. In contrast, decreased expression of *HMT1* was dose-dependently decreased in cells exposed to As. Observed changes in the expression of all genes in 100 pg/mL of As exposed cells were statistically significant (*p*<0.05). However, changes in expression of only three genes (*HDAC7*, *HMT1,* and *HAT1)* were significant (*p*<0.05) in cells exposed to 100 ng/mL of As.

#### 2.2. Effects of chronic exposure to E2 on the expression of histone modifying genes

A dose dependent increase in expression of *HDAC1* and *HAT1*, and a dose-dependent decrease in the expression of *HDAC3, 7* and *HMT1* was observed in cells exposed to 100 pg/mL and 100 ng/mL of E2 (Figure 2). Expression of *HDAC2,* however, was decreased in cells exposed to 100 pg/mL of E2, whereas it was increased in cells exposed to 100 ng/mL of E2. Changes in the expression of *HDAC2*, 3, 7, and *HMT1* were statistically significant (*p<*0.05) in both 100 pg/mL and 100 ng/mL of E2 exposed cells.

#### 2.3. Effects of chronic co-exposure to as and E2 on the expression of histone modifying genes

Interestingly, the expression of all the genes involved in histone modification (HDAC1, 2, 3, and 7, HMT1, and HAT1, that were analyzed in this study were decreased due to co-exposure of As and E2 (Figure 2). This decrease in expression of histone modifying genes in As and E2 co-exposed cells was dose-dependent. Co-exposure with lower dose of As and estrogen was more effective in decreasing the expression of HDAC3, 7 and HAT1, whereas the higher dose was more effective in decreasing the expression of HDAC1, 2 and HMT1. Changes in the expression of all these genes in co-exposed cells were statistically significant (p<0.05).

### 3. Effects of as and E2 on the Expression of Epigenetic Regulatory Proteins and Histone Modifications

**Figure 3:**
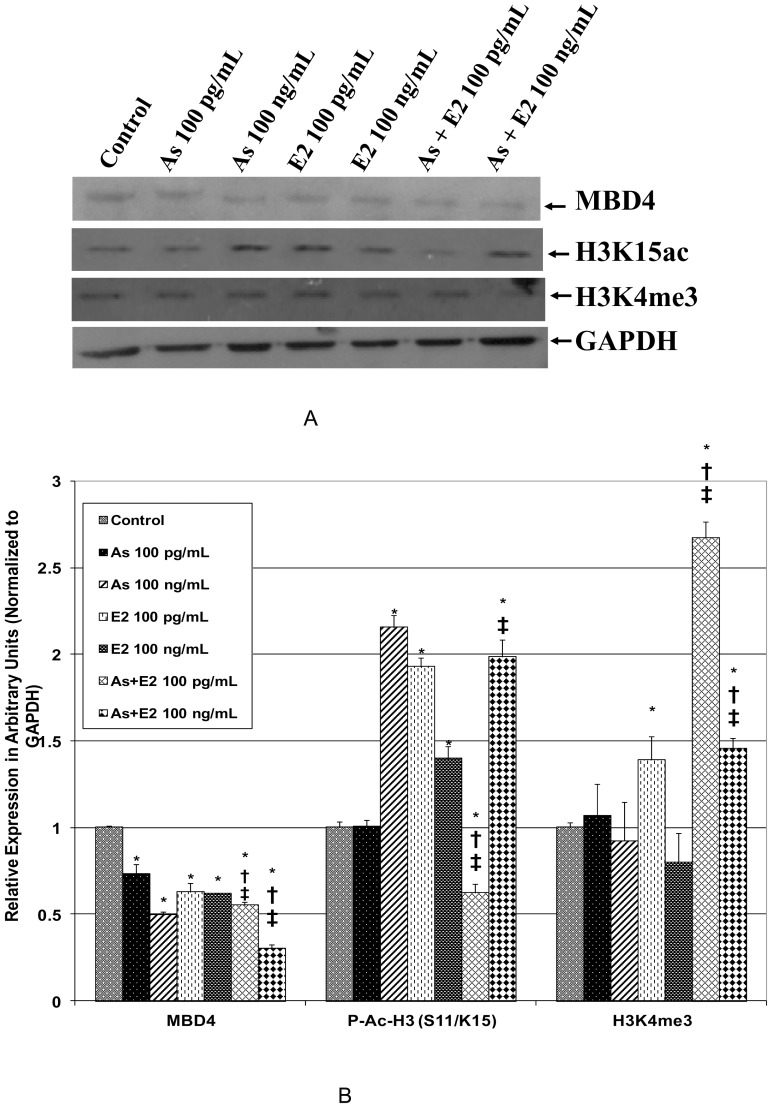
Representative Western blots (3a) and their relative band intensity histograms (3b). Western blots showing the effect of As, E2 and their combination on the expression of MBD4 at protein level as well as the levels of phosphorylated and acetylated histone H3 [p-Ac-histone H3 (S 11/K 15)] and methylated histone H3 [H3K4me3]. The whole cell lysates from As and E2 treated cells were prepared and protein expression/modifications were determined by Western blot analysis as described in Materials and Methods. The histograms represent the signal intensity of protein bands in arbitrary units after normalization with the signal intensity of GAPDH internal control for each sample. The graph represents means of duplicate values. An * indicates significant (*p*<0.05) difference between treatment groups and control. Similarly, † indicates a significant difference (*p<0.05*) between As alone and As in combination with E2. ‡ indicates a significant difference (*p<0.05*) between E2 alone and E2 in combination with As.

Since MBD4 was greatly affected by As and/or E2 exposure at the transcript level, Western blot analysis was performed to further confirm changes in MBD4 expression at the protein level (Figure 3a and 3b). Changes in the levels of p-Ac-histone H3 (S 11/K 15) and methylation (H3K4me3) were also evaluated by Western blot analysis (Figure 3a and 3b). The result of Western blot analysis revealed statistically significant (*p*<0.05) decreased expression of *MBD4* in cells exposed individually to As and E2 as well as their co-exposure. This further confirms the result of quantitative real-time PCR which revealed decreased expression of *MBD4* at transcript level. Western blot analysis also revealed a significant (*p*<0.05) increase in the level of acetylated histone H3 in cells exposed to 100 ng/mL of As, E2, and their combination (Figure 3b). Levels of p-Ac-histone H3 (S 11/K 15) were also significantly (*p*<0.05) increased in cells exposed to 100 pg/mL of E2 but not in 100 pg/mL of As. The cells exposed to 100 pg/mL of As and E2 combination exhibited a significant decrease in the level of p-Ac-histone H3 (S 11/K 15) (p<0.05). The levels of histone H3 methylation as detected by H3K4 me3 antibody were significantly (*p*<0.05) increased in cells exposed to 100 pg/mL of E2.

### 4. Effects of as and E2 Exposure and their Co-exposure on DNA Methylation Patterns

**Figure 4:**
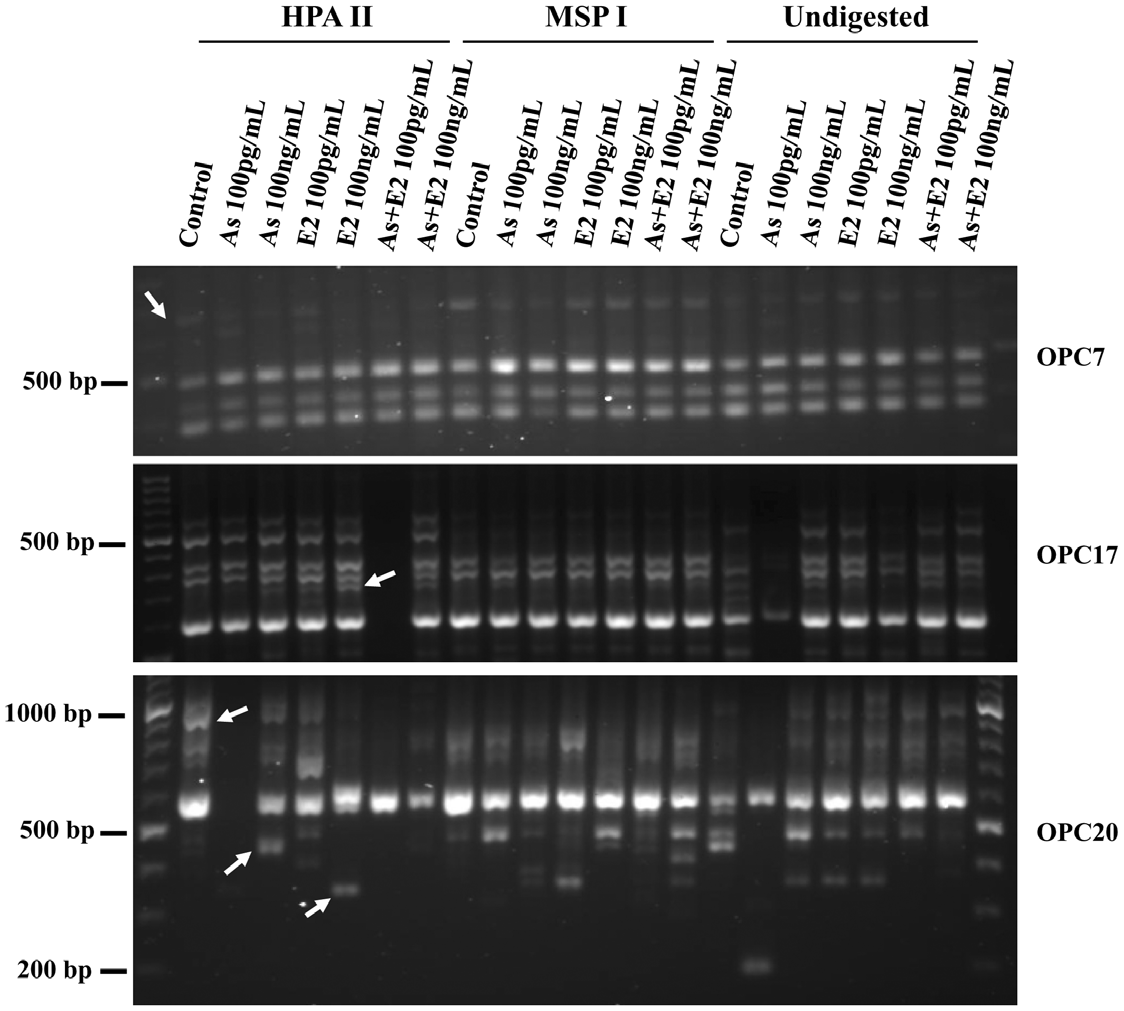
Representative RAPD fingerprints showing DNA methylation changes. Fingerprints generated by primers OPC07 (5′-GTCCCGACGA-3′) [upper panel], OPC17 (5′-TTCCCCCCAG-3′) [middle panel], OPC20 (5′-ACTTCGCCAC-3′) [lower panel] showing the effects of As and E2 treatment alone and in combination on the methylation status in RWPE-1 cells as revealed by loss/gain of amplification products. Genomic DNA isolated from chronically treated RWPE-1 cells was digested with methylation sensitive restriction enzymes (*MspI* and *HpaII*) and then used for RAPD amplifications as described in Materials and Methods section. The loss/gain of RAPD amplification products in the fingerprints from treated RWPE-1 cells are indicated by arrows. Primers used are indicated next to each fingerprint. PCR amplification failed in lane 7 of middle panel and lane 3 of lower panel.

The data of MS-RAPD-PCR revealed that chronic treatment with As and E2 alone and in combination resulted in DNA methylation changes in RWPE-1 cells. Three of ten primers used for MS-RAPD revealed changes in DNA methylation pattern due to As and/or E2 exposure. Representative MS-RAPD fingerprints showing As and/or E2-induced methylation changes are provided in Figure 4. For example, DNA fingerprint obtained by primer OPC17 revealed the presence of 250 bp band from *Hpa*II digested DNA in As and E2 exposed, as well as co-exposed cells. Bands were either absent or hardly detectable in control cells. Bands were also absent in *Msp*I digested DNA, thereby indicating As and E2-induced hypermethylation in these genomic regions (Figure 4). Moreover, there appears to be a dose dependant effect on hypermethylation change as the intensity of this band increased relative to the increased exposure doses of As and E2. Similarly, a 690 bp band in OPC07 was present in control DNA digested with *HpaII* but absent in E2 100 ng/mL as well as in 100 pg/mL and 100 ng/mL combinations of As and E2. This suggests hypomethylation in this genomic region. In the DNA fingerprint using OPC20 primer, a 450 bp band occurred in As 100 ng/mL and a 350 bp band appeared in E2 100 ng/mL samples digested with *HPAII*. Absence of these bands in control suggests hypermethylation in these genomic regions. The DNA fingerprint from *HpaII* digested DNA using primer OPC20 revealed presence of a 900 bp amplification product in control but its absence in As and E2 co-exposed cells indicating hypomethylation in this genomic region.

## Discussion

To our knowledge, this is the first comprehensive study on the effects of As and E2, both alone and in combination, on the expression of epigenetic regulatory genes and their impact on epigenetic marks. Exposure to low levels of arsenic through drinking water is very common in the US population [40], [41]. Clinical and experimental studies have shown the adverse biological effects of arsenic even at the levels below the 10 µg/L limit set by EPA [42]. Given the ubiquitous presence of estrogenic chemicals in the environment, the likelihood of dual exposure to arsenic and estrogen at lower concentrations is very high. However, this environmental concern of dual exposure has not been previously addressed. Additionally the molecular mechanism of adverse biological effects of low level arsenic exposure is not well understood. The novel findings of this study showing epigenetic effects by low levels of arsenic and/or estrogen exposure is highly significant in understanding disease etiology in human populations potentially due to dual exposure of these chemicals.

There are reports on epigenetic alterations in As- induced prostate cancers, however, the mechanism by which global hypomethylation and loci-specific hypermethylation arises is not well understood [7]. Arsenic exposure results in reduction of *DNMT1* and *DNMT3a* mRNA expression as well as their activity [43]. E2 has also been found to down-regulate *DNMT1* and *DNMT3a* during the menstrual cycle [44]. Consistent with these previous reports, decreased expression of *DNMT1* and *DNMT3a* was also observed in As and E2 exposed RWPE-1 cells in the present study. More importantly, as compared to individual exposures, combined exposure to As and E2 resulted in significantly greater reduction in *DNMT1* expression, suggesting a synergistic effect. Results of MS-RAPD showing either global hypo- or hypermethylation in As and E2-exposed cells, further confirmed the consequences of As and E2-induced changes in DNMT expression.

MBD4 plays an important role in apoptotic response through participating in a protein complex with FADD and MLH1 [45]. A decreased apoptotic response in *MBD4* (−/−) mice was observed following exposure to cytotoxic chemicals and gamma radiation [46]. In the present study a significant decrease in the expression of *MBD4* in As and E2 treated cells was observed. Our recently published data as well as the previous report from another group suggest that As exposure causes decreased apoptotic response resulting in increased cell survival [28], [34]. Therefore, the As-induced cell survival and decreased expression of MBD4 in As-exposed cells further corroborates the previous reports on the critical role of *MBD4* in apoptotic response. In addition to the role of MBD4 in apoptotic response, the loss of MBD4 results in many other adverse consequences in the cell [47]. For example, increased mutations at CpG sites (CpG to TpG) and enhanced tumor formation has been reported in MBD4−/− mice [47], [48]. It has also been reported that MBD4 is required for apoptotic induction in DNMT1 depleted embryo [47]. Interestingly, DNMT1 was also decreased in As and E2 exposed cells in this study. Therefore, decreased expression of both MBD4 and DNMT1 in cells exposed to As and E2 and previous reports of As-induced cell survival further provides an epigenetic mechanism for increased cell survival and consequently malignant transformation in prostate epithelial chronically exposed to As.

Changes in histone acetylation have been reported to play an important role in prostate carcinogenesis [23]. There are contradictory reports on histone acetylation changes by arsenic exposure. For example, increased global histone acetylation related to decreased histone deacetylase activity in arsenic exposed HepG2 human liver cancer cells has been reported [34]. However, another study has shown a decrease in histone H3 acetylation due to arsenic exposure [49]. Consistent with the earlier reports by Ramirez et al. [34], our data suggest that arsenic exposure causes increase in histone H3 acetylation and decreased expression of HDACs. Moreover, HDAC inhibitors have also been shown to activate ER-β [50]. Based on these previous reports and current findings, it seems that inhibition of HDACs is essential for E2 to activate ER-β for its biological response of cell growth. Our data also suggests that co-exposure to As and E2 results in increased cell growth and expression of ER-β in RWPE-1 cells (unpublished data). Activation of ER- β by E2 is involved in mitogenic response [50]. Together, these findings suggest that E2 exerts its mitogenic activity in prostate epithelial cells, at least in part, through induction of an ER-β mediated pathway through regulation of HDACs. A significant increase in histone H3 acetylation and its correlation with decreased expression of HDACs in cells co-exposed to As and E2 as observed in present study is consistent with the previous report of arsenic-induced histone H3 acetylation and decreased HDAC activity [34]. We also observed downregulation of HAT1 that should result decreased histone acetylation. In contrast, we observed an increase in p-Ac-histone H3 (S 11/K15). It is not surprising given that K15 acetylation is not regulated by HAT1 [51] and therefore other sites in H3 may be the target for HAT1 mediated acetylation.

The antibody used in this study for H3 detects both K15 acetylation and S11 phosphorylation. Therefore, the increased levels of p-Ac-histone H3 (S 11/K 15) in 100 ng/mL As and 100 pg/mL of E2 exposed cells suggests not only increased acetylation but also H3 phosphorylation at S11. The present finding of increase H3 phosphorylation is consistent with previous reports of As induced H3 phosphorylation [50]. Physiologically, the increased phosphorylation of H3 at S11 is associated with cell cycle upregulation in MAP-kinase dependent manner [52]. This further provides evidence for epigenetic mechanism of As-induced cell survival and growth in RWPE-1 cells [28].

Altered HMT levels have been observed in human tumors [22]. Studies suggest that HMT plays an important role in carcinogenesis. In this study, exposure to E2 at 100 pg/mL as well as the combinations of As and E2 at both 100 pg/mL and 100 ng/mL concentrations resulted in increased level of H3K4me3. Increased levels of histone methylation, H3K4me3, were reported in PC3 prostate cancer cells [53]. Therefore, this study corroborates previous reports and suggests that chemicals such as As and E2 exert their carcinogenic effect by regulation of target genes potentially through histone methylation at H3K4. Interestingly, despite the increase in the level of H3K4me3, expression of HMT1 was decreased in cells exposed to either As or E2 and the co-treatment of cells to these two chemicals. The possible explanation could be that HMTs other than HMT1 might be involved in the observed increase in histone methylation as HMT1 is known to methylate at H3K9me1 and H3K9me2 but not H3K4me3 [54].

Accumulating evidence also suggests that DNA methylation and histone modification are coordinately maintained and these two pathways are dependent on one another [55], [56]. For example, acquisition and/or maintenance of histone methylation at imprinting control regions (ICRs) is mechanistically dependent on DNA methylation in mid-gestation embryos from DNMT3L (−/−) females mice [57]. H3K9 methylation is required for maintenance of global DNA methylation patterns [58]. Presence of H3K4me0-interacting ADD domain in DNMT 3a and DNMT3L suggests an association of DNA methylation reaction with unmodified H3K4 [56]. Experimental evidence also suggests that human DNMT1 plays a critical role in maintenance of histone H3 modification [59]. Treatment with HDAC inhibitors resulting in loss of DNA methylation, further suggest a role of histone deacetylation in DNA demethylation [60]. Based on these evidences for interdependence of DNA methylation and histone modification pathways, it is not unreasonable to suspect that any epigenetic changes of DNA methylation and histone modification induced by As may potentially influence the epigenetic effects of E2 or vice-versa. This may, at least in part, explain some of the epigenetic effects that was either present exclusively or greater in As and E2 combination treated cells than those cells that were given As or E2 treatments individually.

In summary, this study provides novel data on the regulation of genes involved in epigenetic reprogramming that could help in understanding of epigenetic mechanism for As and E2-induced prostate cancer. Additionally, this finding will serve as the foundation for future studies on the epigenetic basis for environmental carcinogen-induced human cancers.
